# Development of a Universal In Vivo Predictive Dissolution Method for a Borderline BCS III/IV Drug Guided by Modeling and Simulations—Acyclovir as a Case Study

**DOI:** 10.1021/acs.molpharmaceut.5c00981

**Published:** 2025-09-22

**Authors:** Mauricio A. García, Fernando Tapia, Benjamín Escares, Peter Langguth

**Affiliations:** † Departamento de Farmacia, Escuela de Química y Farmacia, Facultad de Química y de Farmacia, Pontificia Universidad Católica de Chile, Vicuña Mackenna 4860, Campus San Joaquín, Macul, Santiago 7820436, Chile; ‡ Departamento de Ciencias y Tecnología Farmacéuticas, Universidad de Chile, Santiago 8380494, Chile; § Departament of Biopharmaceutics and Pharmaceutical Technology, 153610 Johannes Gutenberg University Mainz, Mainz 55099, Germany

**Keywords:** in vivo predicted dissolution, virtual bioequivalence, PBPK, small volume dissolution testing, nonsink dissolution

## Abstract

Establishing in vivo predictive dissolution (IPD) conditions requires the consideration of biopredictive aspects during dissolution. For acyclovir, lower dose strengths (200 and 400 mg) can dissolve completely in the gastrointestinal fluids. However, luminal concentrations after administering the highest strength (800 mg) exceed the BCS solubility threshold. Given its poor permeability, sink conditions are not granted for the highest strength. In this study, a universal IPD method for acyclovir tablets was developed using the mini-vessel/mini-paddle apparatus. Computational simulations in a physiologically based pharmacokinetic (PBPK) model further guided the development. Apparatuses with different volumes and stirring conditions were explored, and results served as input for the model. Dissolution of 800 mg of acyclovir tablets in 900 mL of medium largely overpredicted observed plasma profiles due to poor resemblance of nonsink conditions in the lumen. Conversely, dissolution in the mini-vessel filled with 135 mL of HCl, pH 2.0, at 150 rpm, produced accurate predictions of plasma profiles, without affecting previous successful predictions with the lowest strength tablets. Furthermore, in-human and virtual bioequivalence studies confirmed the predictive potential of this method. Therefore, the aforementioned dissolution conditions can be considered as a universal IPD method for acyclovir immediate-release tablets.

## Graphical abstract

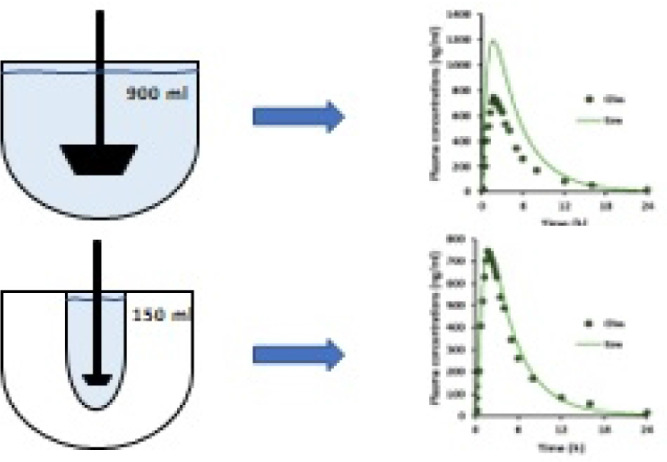

## 1 Introduction

Modeling and simulations have become attractive tools for the pharmaceutical industry in the past few years. Accordingly, pharmacokinetic (including PBPK and PBBM) models can be used for guiding drug design, understanding mechanisms of drug disposition, and studying drug–drug interactions, among many other applications. Recently, these models have also been used to inform the drug development process, called model-informed drug development (MIDD).^1^ In this framework, modeling can be used to select more promising formulations, setting dissolution specifications, defining dissolution safe space, etc.^2–6^ One important application relates to the development of in vivo predictive dissolution (IPD) methods.^7^ These can anticipate drug product performance from in vitro testing,^7^ hence the interest of pharmaceutical industry in these methods. Furthermore, IPD testing is even more convenient if a simple, robust, and generalizable method can be developed, which can be implemented for routine tests, e.g., quality control (QC) tests.

Development of an IPD method may consider biologically relevant features as the starting point. For instance, media buffer molarities have been lately modified to design biopredictive dissolution methods for poorly soluble ionizable drugs.^8^ Accordingly, for a poorly absorbed drug, the lack of a physiological sink increases the relevance of dissolution on oral pharmacokinetics. Acyclovir is a model poorly permeable drug that exhibits a fraction absorbed between 10% and 30%.^9^ However, whether it is completely soluble in gastrointestinal fluids depends on the dose strength administered. Acyclovir’s lowest solubility in biological fluids is 2.33 mg/mL, at a pH of 5.8,^10^ and the highest dose strength is 800 mg, commercially available as immediate-release (IR) tablets.^9^ Considering a glass of 250 mL of water, the dimensionless Do number (equal to the ratio between dose/volume and drug aqueous solubility) is 1.37,^11^ thus BCS class IV.^12^ However, it can be argued that dissolution may be complete for lower strengths (200 and 400 mg),^9^ where Do values are calculated to be 0.34 and 0.69, respectively.

In a previous study, acyclovir plasma profiles of lower dose strengths (200 and 400 mg) were successfully predicted by combining dissolution in the USP type II apparatus with PBPK modeling.^13^ In that article, dissolution input was obtained from experiments in 900 mL of media volume. For those lower strengths, physiological sink is granted by the low acyclovir mass and its sufficient water solubility, explaining why the duration of dissolution was extremely rapid, and thus demonstrated to be negligible compared to a solution.^13^ However, this may not be the case for the highest strength of 800 mg. In fact, recent measurements of gastric water volume show that the resting volume is only between 25 and 35 mL.^14,15^ This can rise up to nearly 300 mL upon water ingestion, but it is rapidly emptied within 30 min.^15^ On the other hand, small intestinal water volume has been determined to amount to an average of 82 mL, with a high variability (±65 mL).^16^ The small gastrointestinal volume, together with acyclovir’s poor permeability, questions the assumption of sink conditions for the dissolution of the 800 mg dose strength when formulated as IR tablets.

One standardized, though noncompendial, apparatus is the miniaturized version of the USP type II apparatus (the mini-paddle). The 250 mL mini-vessel has been used to study drug dissolution from several products in the market.^17,18^ However, there is a smaller version for 150 mL that has greater potential to mimic the small physiological volume (Figure S1). Accordingly, dissolution media are stirred by a suitable mini-paddle similar to the standard apparatus II. This device has been both experimentally and theoretically characterized for its use as a dissolution apparatus.^19–21^

Therefore, we hypothesized that a smaller volume may be more physiologically relevant for studying the dissolution of the 800 mg acyclovir dose strength. In this work, the dissolution of the highest and the lowest acyclovir strengths, 200 and 800 mg, was studied with the mini-paddle and the profiles were subsequently used as input into the PBPK model previously developed and validated.^13^ Furthermore, the effect of the mini-paddle stirring rate and volume on dissolution profiles was optimized in order to develop a one-size-fits-all IPD method for acyclovir, across commercial dose strengths. Finally, simulation outputs were compared with observed plasma profiles to inform the development of the IPD method. The IPD conditions consisted of the mini-vessel apparatus filled with 135 mL of hydrochloric acid media, pH 2.0, at 150 rpm. This method was supported by both in vivo evidence and computational simulations.

## 2 Materials and Methods

### 2.1 Materials

Hydrochloric acid was purchased from Merck (Sigma-Aldrich, Merck KGaA, Darmstadt, Germany). Acyclovir (IUPAC) was purchased from AmBeed (Arlington Heights, IL 60004, USA). Acyclovir 200 mg IR tablets, reference product: Zovirax 200 mg, were used. Furthermore, four acyclovir 800 mg IR tablets available in Chile were tested, including the reference: Zovirax 800 mg and three generic brands (Products A, B, and C, respectively). While products A and C were labeled as bioequivalent to the reference, there was no information about the bioequivalence status of product B. All tablets were acquired from local pharmacies.

### 2.2 Bioequivalence Trial

Data of bioequivalence for acyclovir 800 mg IR tablets (product A versus the reference) were kindly provided by the sponsor. Briefly, an open label, randomized, two treatment, two sequence, two period, single dose, crossover, and bioequivalence study was performed in 42 healthy male volunteers (study number: AZ/BE/03/16/12). Subjects (average age = 31.1 years; height = 168.7 cm; and body weight = 68.2 kg) were administered with either the reference or the product A and blood samples were withdrawn at 0, 0.25, 0.5, 0.75, 1, 1.25, 1.5, 1.75, 2.0, 2.25, 2.5, 2.75, 3, 3.5, 4, 5, 6, 8, 12, 16, and 24 h. Plasma was isolated, and plasma acyclovir was quantified by HPLC Ms/Ms.

### 2.3 In Vitro Dissolution

#### 2.3.1 USP Type II Apparatus

Acyclovir dissolution (*n* = 3) was tested using the USP type II apparatus (Classic 6 Dissolution Tester, Teledyne Hanson Research, Chatsworth, CA) at 50 rpm, 37 °C. Tablets were submerged into 900 mL of l 10 mM HCl media, pH 2.0, and 5 mL samples were taken at 5, 10, 15, 20, 30, 45, and 60 min, and afterward every 30 min until 120 min. The media were refilled immediately after each sampling. Samples were filtered (PTFE, 0.45 μm), diluted, and analyzed by spectrophotometry at λ = 250 nm.

#### 2.3.2 Mini-Vessel Apparatus

Alternatively, acyclovir dissolution (*n* = 3) was studied using the mini-paddle, a miniaturized version of apparatus II (Teledyne Hanson Research, Chatsworth, CA). Briefly, the dissolution tester was adapted with the mini-vessel and mini-paddle (Figure S1). Experiments were conducted in the same media (HCl, pH 2.0) as those performed with the USP type II apparatus at 37 °C. The effect of stirring rate on tablet dissolution was studied by setting the mini-paddle rotational speed at 100, 125, or 150 rpm. Furthermore, the effect of the volume was tested by conducting experiments in either 135 or 150 mL. The sampling scheme was the same as aforementioned but reducing the sampling volume to 1 mL. Filtering and analytics were kept unaltered.

### 2.4 PBPK Model

The PBPK model that was previously built for acyclovir IR tablets in GastroPlus V. 9.8.1 (Simulations Plus, Inc., Lancaster, CA)^13^ was adapted to software version 9.8.3 with slight modifications: (i) ADH-mediated metabolism was removed due to its negligible contribution to plasma concentrations;^13^ (ii) The default aqueous diffusion was slightly increased from 8.9 × 10^–6^ to the experimental value 9.24 × 10^–6^ cm^2^/s, as recently reported by our working group;^22^ (iii) The optimized effective permeability (*P*_eff_) value of 2.9 × 10^–5^ was changed to 3.56 × 10^–5^ cm/s based on our recent in vitro determinations using MDCK cells and an in-house built calibration set (number of compounds = 5, *r* ^2^ = 0.9692, unpublished data); and (iv) *V*_max_ values of renally expressed transporters, OAT2 and MATE1, were slightly increased to 0.12 and 0.024 mg/s/mg of transporter, respectively, to match the plasma profiles reported in our previous work.^13^ Given the uncertainty of the in vivo values for transporter *V*_max_, the aforementioned modifications of drug elimination transporter-mediated kinetics were necessary to compensate for the changes in diffusion and permeability, which affected absorption kinetics. The updated table of input parameters is given in Table S1. The model was validated against plasma profiles reported by Vergin et al.^23^ and Al-Yamani et al.^24^ in the same fashion as previously reported^13^ (Figure S2).

### 2.5 Simulations

#### 2.5.1 Single Profile Simulation

In vitro dissolution fitting to the Weibull function was carried out with the controlled release module available in GastroPlus (eq 1).

1 $$W_{diss}=W_{max}\left(1-e^{\left(-\frac{{\left(t-t_{lag}\right)}^{\beta }}{\alpha }\right)}\right)$$

Where *W*_diss_ is the percentage dissolved at time = *t*, and α and β are typical Weibull parameters for time-scaling and shape, respectively. In this model, the lag time (*t*_lag_) was set to 0, as is usually done for IR formulations, while the maximum percentage released (*W*_max_) was fixed to a value calculated from eq 2:

2 $$W_{max}=\frac{S_{pH\;2.0}\times V\times 100}{D}$$

Where *S*_pH 2.0_ = 3.6 mg/mL is acyclovir solubility at pH 2.0 and 37 °C,^10^ *V* is the dissolution media volume in the mini-vessel (either 135 or 150 mL), and *D* is the acyclovir dose strength (800 mg). Fixing these parameters reduced the risk of overfitting. Finally, the α and β parameters were fit to the dissolution data.

Simulations for each formulation were run from respective Weibull curves into the PBPK model by using the “CR: Dispersed” dosage form selection. Simulation outputs, single plasma-time profiles, were compared to observed plasma profiles by means of the prediction error (PE) on the pharmacokinetic parameters *C*_max_ and AUC_0–inf_.

#### 2.5.2 Virtual Bioequivalence

A virtual population of 42 male subjects was created in the PEAR module to match the population of the bioequivalence trial (see above). Similar settings as in the previous study were used,^13^ but the CV of *P*_eff_ was set to 35% to match within-subject variability in the study (Table 1). Ten runs of crossover virtual bioequivalence trials were conducted on this population using respective Weibull input to characterize each treatment. Data were analyzed using standard bioequivalence metrics.^25^

**Table 1:** Bioequivalence Outputs between Product A and the Reference

Statistics	*C*_max_	AUC_0–t_	AUC_0–inf_
Geometric mean T/R ratio (%)	105.12	101.15	95.55
Lower limit 90% CI	92.98	88.87	85.48
Upper limit 90% CI	118.85	115.12	106.8
Within subject CV (%)	34.37	36.33	31.00

## 3 Results

### 3.1 Bioequivalence of Acyclovir 800 mg IR Tablets

Oral bioavailability of 800 mg of acyclovir IR tablets was compared between product A and the reference in a bioequivalence trial. All subjects (*n* = 42) completed the study, with both products being well tolerated. Product A was deemed bioequivalent to the reference (Table 1).

### 3.2 Dissolution in Compendial USP Type II Apparatus

Both products, A and the reference, were also characterized for their in vitro dissolution in 900 mL of HCl media, pH 2.0 (Figure 1A). Under these conditions, both products reached 100% dissolution, although dissolution was faster for the reference compared to product A. When using these data as direct input into the PBPK model, simulations revealed that dissolution inputs largely overpredict observed plasma profiles (Figure 1B,C). In fact, both *C*_max_ and AUC_0–inf_ were overestimated by 1.49–1.67-folds and 1.48–1.65-folds, respectively. This PBPK model already demonstrated its predictive potential for lower acyclovir strengths (200 and 400 mg), where sink conditions are granted.^13^ Thus, these findings suggest that a 900 mL volume may be too large to represent the loss of sink conditions in the lumen for the highest dose strength. Therefore, the minivessels were used to better mimic the small water availability throughout the gastrointestinal tract.

**Figure 1:**
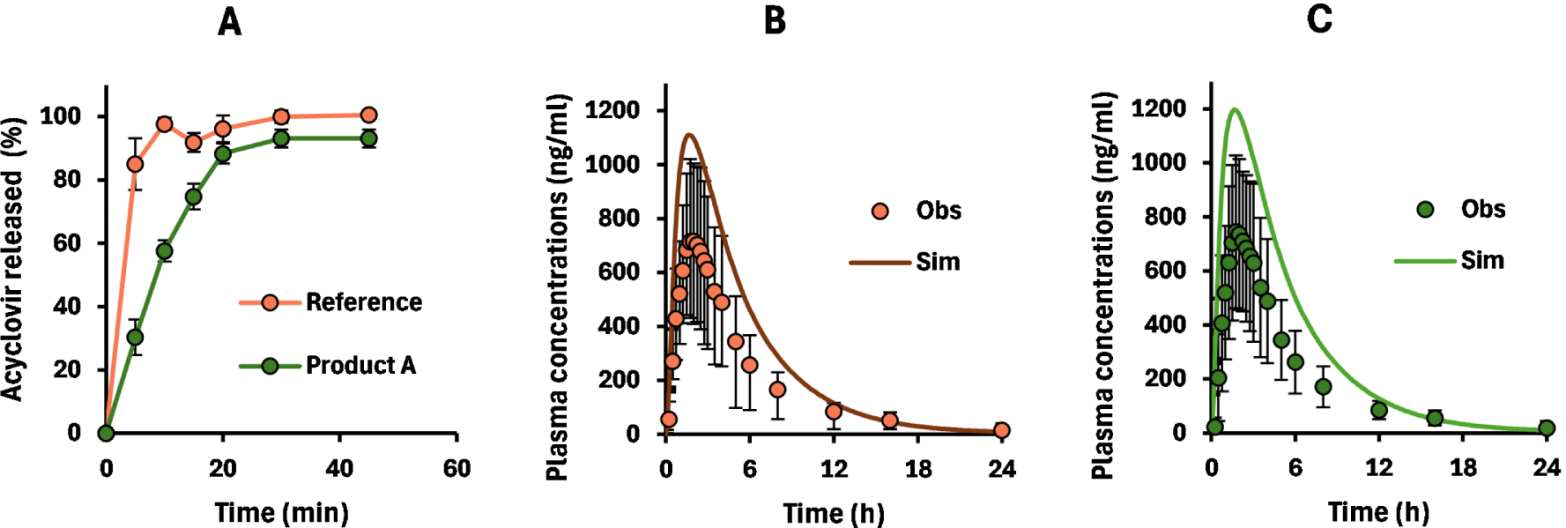
Dissolution of the reference and product A (*n* = 3) in the USP type II apparatus filled with 900 mL of HCl (A). Panels B and C, respectively, show observed profiles from the bioequivalence trial (*n* = 42) for the reference (orange) and product A (green). Simulations using dissolution in panel A as input are shown in solid lines.

### 3.3 Effect of Dissolution Volume and Rotational Speed

This work aimed at developing a new universal method for testing in vivo predictive dissolution (IPD) of oral acyclovir products across marketed dose strengths. It was necessary for the mini-paddle apparatus to perform as well as the older method (i.e., the classical USP type II apparatus) performed for the lower strengths. First, in vitro dissolution of acyclovir 200 mg IR reference tablets was studied with the mini-paddle under different experimental conditions (Figure 2A). As expected, the acyclovir dissolution rate increased with higher stirring intensity (rpm), releasing more than 95% of the drug across experimental conditions. On the one hand, this fact demonstrates that complete dissolution of acyclovir 200 mg can be rapidly achieved in small volume, suggesting sink conditions are granted. This was also consistent with solubility values previously reported by Shojaei et al.^10^ On the other hand, with *t*_max_ being at around 1.75–2 h (Table S2), these results also suggest that differences within this time frame may impact plasma concentrations. This was confirmed with plasma curve simulations (Figure 2B).

**Figure 2:**
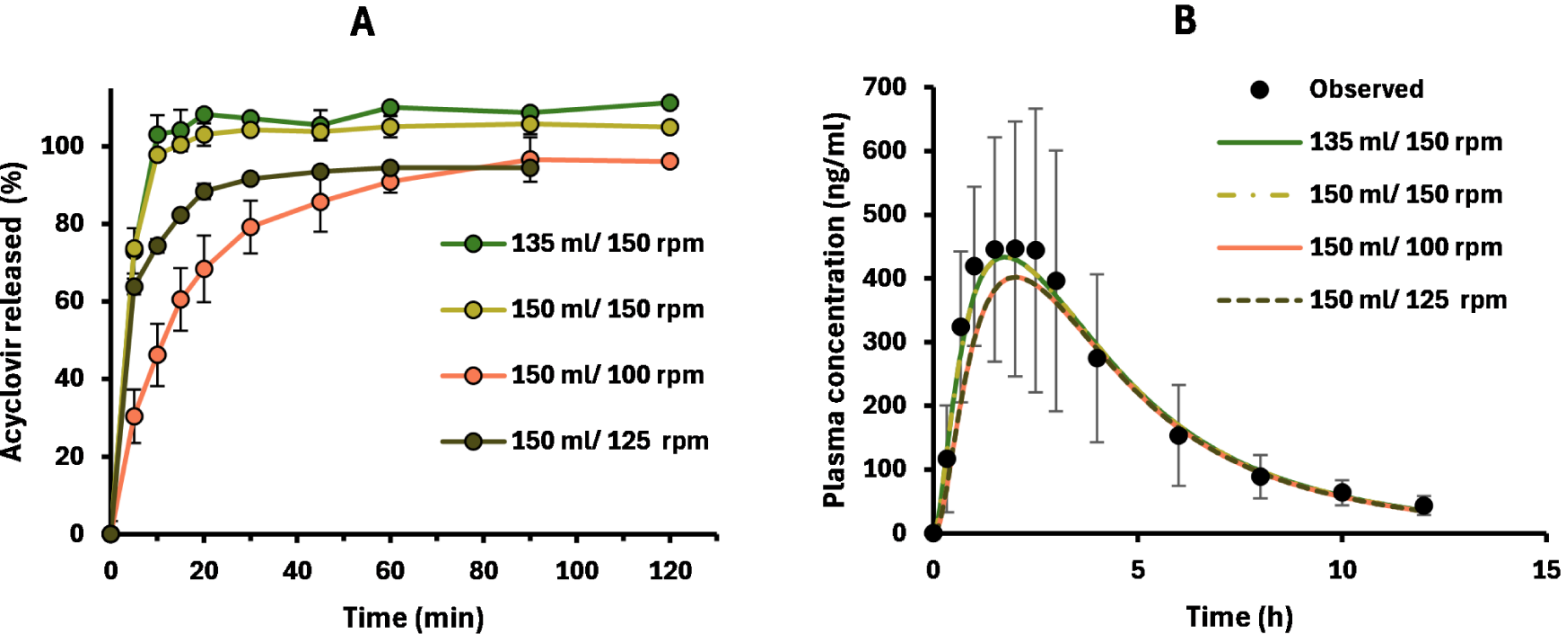
Dissolution profiles of acyclovir 200 mg reference immediate-release tablets (*n* = 3) in the mini-vessel under different experimental conditions (A). Simulations were run from those profiles and compared to data published by Vergin et al.,^23^ with the same product (B). Error bars represent the SD.

Because of the good performance of the mini-paddle for the 200 mg dose strength, the in vitro dissolution of acyclovir 800 mg (product A and the reference) was further studied in this setup. Figure 3A,B displays the effect of rpm on the dissolution profile in 150 mL of media. Overall, drug dissolution was incomplete, with the maximum amount (*W*_max_) dissolved ranging from 67.7% to 75.5%. This was in good agreement with the value calculated in 150 mL of media (*W*_max_ = 67.5%). For the reference product, all profiles reached the *W*_max_ before the end of the experiment. Conversely, the dissolution of product A at 100 rpm only reached a 43.8% of drug dissolved after 2 h. Furthermore, results suggest that the dissolution rate reaches a maximum above the 125 rpm for the reference, as no noticeable difference was observed between profiles at 125 and 150 rpm. By contrast, dissolution profiles of product A were overall slower than the reference, such that the maximum dissolution rate was not reached at 125 rpm. Therefore, experiments performed in 150 mL at 125 rpm (method: 150 mL/125 rpm) seemed to allow an appropriate discrimination between profiles. Moreover, experiments at 150 rpm suggest this rotational speed may be more appropriate to obtain similar dissolution profiles between the products.

**Figure 3:**
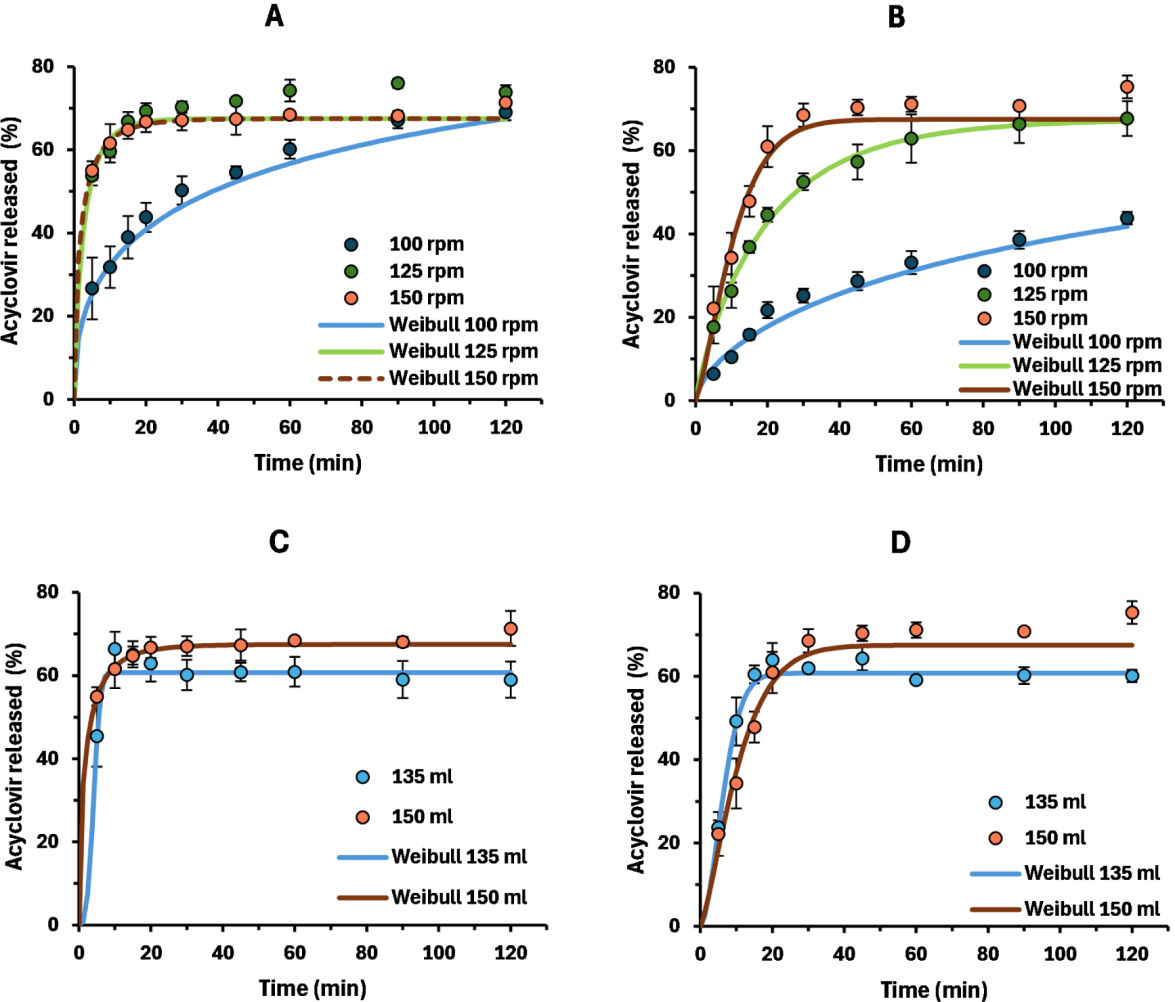
Dissolution profiles of acyclovir 800 mg immediate-release tablets (*n* = 3) from the reference (A and C) and product A (B and D). Panels A and B show the effect of rotational speed using 150 mL of dissolution media, while panels C and D display the effect of medium volume at 150 rpm. Lines represent the Weibull fitting to the dissolution data. Error bars represent SD.

Following, the effect of dissolution volume was assessed while keeping the rotational speed at 150 rpm. Figure 3C,D depicts that decreasing the volume to 135 mL did not drastically affect the dissolution rate for either of the products. This was caused by the rapid mini-paddle rotational speed. Nonetheless, the amount released under these conditions (method: 135 mL/150 rpm) was lower than in 150 mL and consistent with the *W*_max_ = 60.75% calculated with eq 1.

Dissolution profiles obtained with the aforementioned methods were fit to Weibull (Figure 3) and used as input in the model to simulate 800 mg of acyclovir plasma curves of each product (Table 2). As expected, the slow release at 100 rpm led to large average prediction errors (PEs) of −17.1% and −21.3% in AUC and *C*_max_, respectively. These values are far from the typical average PE limit of 10%.^26^ This underprediction was particularly evident for product A due to its much slower dissolution. In contrast, the 150 mL/150 rpm method resulted in a slight overprediction of *C*_max_, with an average PE of 11.4%. Considering that this overprediction was not seen with the 135 mL/150 rpm method (Table 2), it was most likely caused by the total amount released. In fact, Figure 4 shows that the method produced a good prediction of plasma profiles for each product. Similarly, the 150 mL/125 rpm method also produced acceptable PE values (Table 2), although it showed to slightly overestimate *C*_max_ of the reference (Figure 4). Therefore, the performance of these two methods was tested in further studies.

**Table 2:** Prediction Errors Obtained with the Mini-Paddle^a^

	150 mL/100 rpm		150 mL/125 rpm		150 mL/150 rpm		135 mL/150 rpm	
	*C*_max_	AUC	*C*_max_	AUC	*C*_max_	AUC	*C*_max_	AUC
**Reference**	–1.0	3.6	12.2	8.5	12.1	8.4	0.79	–2.94
**Product A**	–41.7	–37.9	4.1	1.2	10.7	6.4	–3.62	–5.27
**Average**	–21.3	–17.1	8.2	4.8	11.4	7.4	–1.42	–4.10

**Figure 4:**
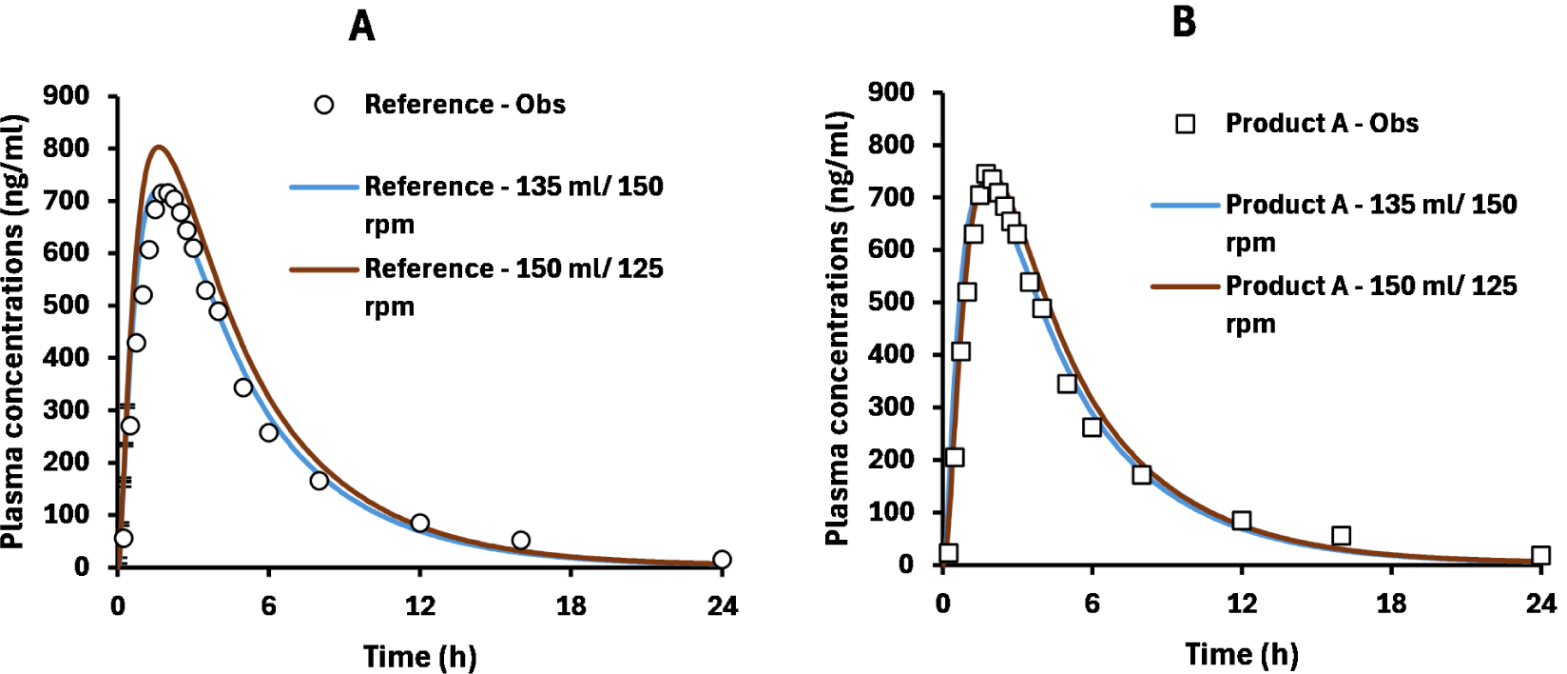
Observed plasma concentration-time profiles for the reference (A) and product A (B) are shown in circles and squares, respectively. Blue and brown lines show simulation runs with dissolution methods: (i) 135 mL/150 rpm or (ii) 150 mL/125 rpm, respectively.

^a^AUC_0–inf_ was used for calculations.

In vitro dissolution of two additional brands of acyclovir 800 mg, products B and C, was further tested with these methods. Figure 5 shows a comparison of dissolution profiles for every product investigated in this article using the 135 mL/150 and 150 mL/125 rpm methods, in panels A and B, respectively. While the bioequivalence of the product C has already been demonstrated (product package labeled as bioequivalent), the absence of labeling for the product B means that its bioequivalence is unknown.

**Figure 5:**
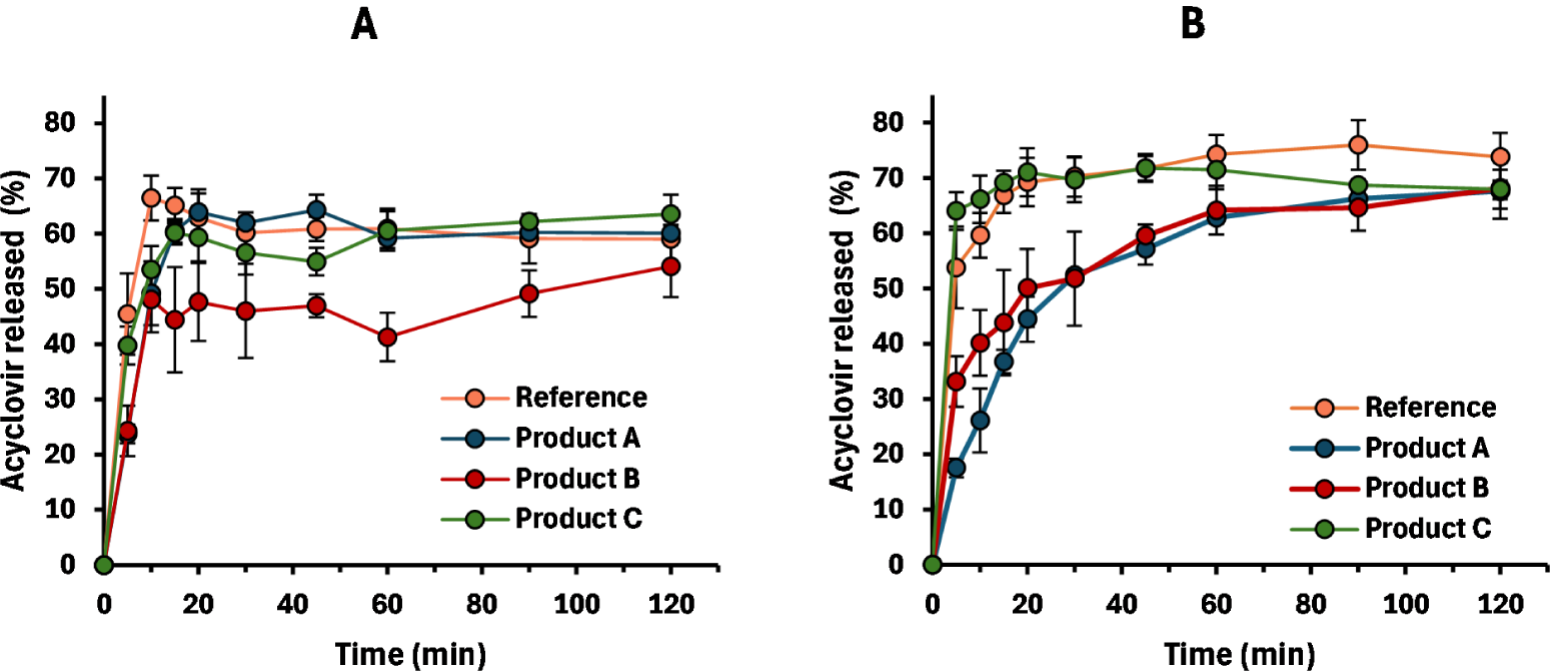
Dissolution of the reference (orange), product A (blue), product B (red), and product C (green) using the 135 mL/150 or 150 mL/125 rpm method, panels A and B, respectively (*n* = 3). Error bars show SD.

### 3.4 Virtual Bioequivalence

Ultimately, the dissolution profiles in Figure 5 were fit to their respective Weibull functions (Table S3) and incorporated in the model to run virtual bioequivalence (VBE) trials (Table 3). The VBE analysis showed that the 135 mL/150 rpm method correctly predicted the bioequivalence of products A and C to the reference in 100% of the virtual trials. Meanwhile, the 150 rpm/125 rpm method was capable of detecting the bioequivalence of product A in only 50% of the trials, below the 80% threshold previously proposed.^2^ Examples of VBE simulations are shown in Figure 6. Therefore, in light of our findings, the 135 mL/150 rpm experimental conditions were considered optimal to develop a universal IPD method for acyclovir IR tablets. The universality of the IPD method was further confirmed by testing in vitro dissolution of 200 mg strength products used in our previous work,^13^ and conducting single profile simulations (Table S4). Prediction errors for *C*_max_ and AUC in those simulations were always below 10%.

**Table 3:** Virtual Bioequivalence Outputs for All Products Are Compared to the Reference

	Percentage of VBE passed out of 10 virtual trials (pass or fail decision)		
	Product A	Product B	Product C
**135 mL/150 rpm**	100% (pass)	0% (fail)	100% (pass)
**150 mL/125 rpm**	50% (fail)	40% (fail)	100% (pass)

**Figure 6:**
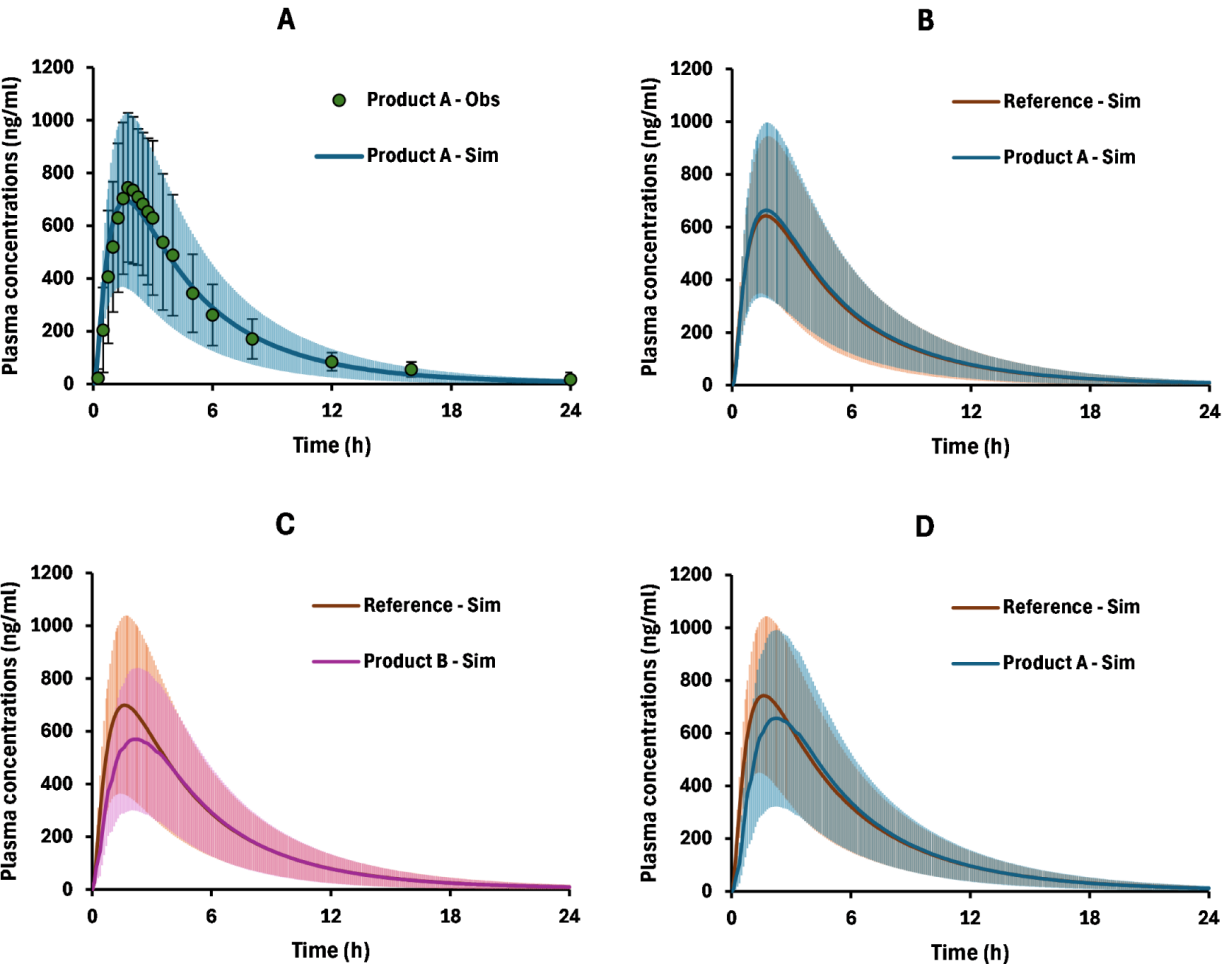
Examples of results from population simulation (*n* = 42). Panel A shows a comparison between observed and simulated plasma curves in the simulated population after administering product A with the 135 mL/150 rpm method (VBE trial 01). Panel B shows the VBE trial 06 between product A and the reference with the 135 mL/150 rpm method, an example of passing the VBE test. Panel C displays an example of failed VBE (trial 01) between product B and the reference using the 135 mL/150 rpm method. Panel D exhibits an example of not passing the VBE between product A and the reference as a consequence of using the 150 mL/125 rpm method (VBE trial 10). Colored contours represent the SD.

## 4 Discussion

The present study aimed at developing a universal IPD method for the challenging BCS III/IV borderline drug, acyclovir. Typical acyclovir dose strengths in the market include 200 and 400 mg, as IR tablets.^9^ However, the 800 mg dose strength is available in the market in several countries,^9^ which is in line with the maximum recommended single therapeutic dose.^12^ In a previous study, we developed and validated a PBBM approach for lower acyclovir strengths (200 and 400 mg). In that work, in vitro dissolution profiles obtained in the USP type II apparatus filled with 900 mL of hydrochloric acid or phosphate as dissolution media were used as input.^13^ When dissolution of the highest strength in that large volume was studied, plasma profiles were highly overpredicted (Figure 2). This is because of both solubility and permeability. On the one hand, acyclovir solubility in gastric media (HCl, pH= 2.0) is 3.6 mg/mL,^10^ so the volume needed for dissolving 200 mg (D/S) is 55.6 mL. This value is sufficiently small for sink conditions to be granted throughout the gastrointestinal tract. On the other hand, the volume for dissolving 800 mg is 222.2 mL, which is 2- to 3-folds larger than the volume of gastrointestinal fluids.^14–16^ Even though drug product intake with 250 mL of water is recommended, this volume is rapidly absorbed in the fasting conditions typically used in bioequivalence studies.^14,27^ For typical low-solubility drugs, i.e., BCS class II, physiological sink is granted by transepithelial permeation.^11^ However, this is not the case for a poorly permeable drug such as acyclovir. Therefore, a dissolution method involving reduced media volume was investigated.

### 4.1 In Vitro Considerations

In this manuscript, the noncompendial miniaturized version of the type II apparatus (mini-paddle) was utilized for mimicking small gastrointestinal volume. Advantages of this setup include its low cost (as it can be easily adapted to a conventional USP type I/II dissolution tester) and standardization (as commercial versions are available). Furthermore, this apparatus was particularly advantageous because it can simulate the in vivo lack of a sink, which is essential to develop an IPD method for a BCS IV compound. The findings in this article show that IPD conditions for acyclovir dissolution in the mini-vessel were 135 mL of HCl media (pH 2.0) at 150 rpm (Table 3). While small volumes and acidic media resemble some biorelevant features, the rotational speed of 150 rpm may seem too fast at a first glance. However, mechanical stress caused by peristalsis may justify this agitation.^28^ Further, experimental and computational studies have compared hydrodynamics between the mini-vessel and the apparatus II. For instance, Scheubel et al. showed that dissolution profiles of IR tablets obtained in the mini-vessel at 150 rpm were comparable to curves from experiments in the USP type II apparatus filled with 900 mL at 100 rpm.^21^ Consistently, computational fluid dynamic (CFD) simulations suggested that an agitation speed of around 130 rpm in the mini-vessel was equivalent to 100 rpm in the conventional USP type II device.^20^ Those findings were experimentally supported by particle image velocimetry measurements.^19^ Therefore, the hydrodynamics generated by the rotational speed of 150 rpm, as proposed in this study, may not be excessively fast and are expected to be within ranges used in 900 mL of dissolution testing. Even though the method developed in this article suits nicely the case of acyclovir, generalization of these conditions for all BCS III/IV drugs demands further testing with other drugs. However, the success of the approach presented here suggests that it may be a suitable starting point for developing IPD for other borderline compounds.

A compendial alternative to performing in vitro dissolution in small volume is the flow-through cell (USP type IV) apparatus.^29^ In that apparatus, the dosage form is in contact with a small volume of media in the cell and dissolved drug is removed by convectional fluid flow. That fluid flow creates a sink in the test (by convection) that is different from the physiological sink, caused either by high API solubility or because of transepithelial diffusion (permeation). Thus, apparatus IV may not fully capture the essence of the in vivo sink, and flow rates have to be usually set to low values, leading to extremely slow profiles. In fact, while apparatus IV was successfully used in the design of biologically relevant dissolution methods for some low-solubility BCS II drugs, these studies involved time-scaling functions to correct differences between in vitro and in vivo predicted outputs through correlations.^30–32^ Recently, the USP type IV was used to develop a biopredictive dissolution method for extended-release tacrolimus capsules, where in vivo dissolution is expected to be slow, as well.^33^ Nonetheless, this is not the case for acyclovir 800 mg IR tablets, as demonstrated by its short *t*_max_ ranging from 1.75–2 h (Table S2). Consequently, the mini-vessel resembles better dissolution conditions for formulations used in this study: immediate-release BCS class IV. Remarkably, acyclovir dissolution profiles obtained with the mini-paddle led to accurate predictions of plasma profiles without needing any time-scaling approach (Figure 4). This method not only improved in vivo predictions for the highest strength, but it also did not significantly impact drug product performance prediction for the 200 mg strength (Figure 2 and Table S4). Unfortunately, the lack of access to in vivo data for the middle dose strength (400 mg) prevented us from testing our IPD method on that strength. However, considering the IPD successfully predicted plasma profiles for both the highest and lowest dose strengths, it is reasonable to expect that it would be suitable for the middle dose strength too. All together, these facts further supported the approach proposed in this paper.

### 4.2 In Silico Considerations

One aspect worth discussing is the dissolution model applied to run simulations. In our previous work, dissolution data were directly incorporated into the software to run simulations.^13^ This approach was chosen because of its previous success when incorporating dissolution data from IR high-solubility drugs.^34^ This is, however, not necessarily the case for the highest acyclovir strength. Therefore, this review explored the use of an alternative method. While the first-order kinetic model is widely used for IR dosage forms, this model may be too simple to describe dissolution profiles in this work. In fact, the Weibull function collapses into the first-order model when both conditions, *t*_lag_ = 0 and β = 1, are met. In this regard, Table S3 demonstrates the need of using Weibull, instead of the simpler first-order model, as β values differed (sometimes between 3- and 4-folds) from the unity. Moreover, this method did not improve prediction performance for the 200 mg dose strength (Table S4). Furthermore, the empirical nature of the Weibull function maintains the weight of the prediction on the in vitro dissolution data, thus supporting the in vivo predictive potential of the in vitro method.

Recently, a group of articles have been published, where the best practices for PBBM, including VBE trials, have been thoroughly discussed.^2–5^ Simulations carried out in this study overall considered those aspects. For instance, it is well recognized that a large sample size (i.e., number of subjects) increases the likelihood of passing the bioequivalence.^5^ That is why the recommendation was to use a clinically relevant sample size.^2,5^ Accordingly, the virtual population generated in this study consisted of 42 male subjects who matched the biometric parameters of the population that participated in the actual bioequivalence trial. Another aspect discussed was within subject variability (WSV). Heimbach et al. suggested to propagate WSV by modifying the CV for relevant parameters when creating the virtual population.^5^ Successful case studies implementing this strategy were recently published.^35^ In fact, this strategy was previously applied by introducing a CV of 25% in the *P*_eff_ parameter to match acyclovir plasma profiles reported in the literature.^13^ Nonetheless, this study reported the actual WSV values (around 35%, Table 1), which were consistent with a highly variable drug like acyclovir. Hence, this CV was applied to *P*_eff_ to generate the virtual population by Monte Carlo simulations. Finally, previous VBE studies usually simulate multiple runs before reaching a conclusion.^36,37^ A total of 10 runs with a success ratio of 80% or higher has been considered reasonable to get confidence in the VBE outcomes.^2^

In this study, we tested the reference, two products bioequivalent to the reference (A and C), and one product with unknown bioequivalence status (product B). While the IPD method coupled with VBE trials successfully predicted the bioequivalence of products A and C, the 150 mL/125 rpm method was only able to predict the bioequivalence of product C (Table 3). The latter method failed the VBE test for product A in 5 out of 10 trials, all of them due to both *C*_max_ and AUC being below the lower limit for the 90% confidence interval (CI). On the one hand, the 150 mL/125 rpm method assessed the kinetic contribution of dissolution, by being able to discriminate between the reference and product A in terms of dissolution rates (Figure 3). This was consistent with failures in the absorption rate parameter, *C*_max_. However, input dissolution curves for both products reached the same *W*_max_ = 67.5% (fixed) throughout the assay. Therefore, the failures associated with AUC might be explained by the relatively short acyclovir absorption window^13^ (Table S2). On the other hand, the weight of the IPD method relied mainly on the contribution of the thermodynamic solubility. With both products being rapidly dissolved until reaching the *W*_max_ of 60.75%, the IPD method was able to successfully predict acyclovir PK (Figure 4) and product bioequivalence (Table 3). It is worthwhile to notice that the IPD and 150 mL/125 rpm produced very similar single simulations for product A (Figure 4B), although the *W*_max_ values differed by around 7%. This fact suggests that differences in fraction absorbed depend mainly on how fast the amount of drug is available to be absorbed. For instance, the simulation with the reference showed that 540 mg of acyclovir was rapidly released in the first 30 min when using the 150 mL/125 rpm method (Figure 7A). That amount dissolved remained constant and only decreased after the third hour of simulation. By contrast, product A released only 412 mg of acyclovir in the first 30 min when using the same method (Figure 7B). Even though the amount dissolved slowly increased until reaching a peak of 540 mg dissolved at 3 h, this difference was sufficient to reduce the acyclovir plasma peak from 802 ng/mL (reference) to 745 ng/mL (product A), Figure 7A,B, respectively. When using the IPD method on product A, the amount dissolved rapidly reached 485 mg and it was kept during the next 3 h of simulation (Figure 7C). Given the similarity between simulated plasma profiles with the IPD and the 150 mL/125 rpm method (Figure 4), this observation reveals that total acyclovir dissolved is irrelevant if it is not rapidly released. This conclusion is also consistent with the absorption window previously proposed for acyclovir.

**Figure 7:**
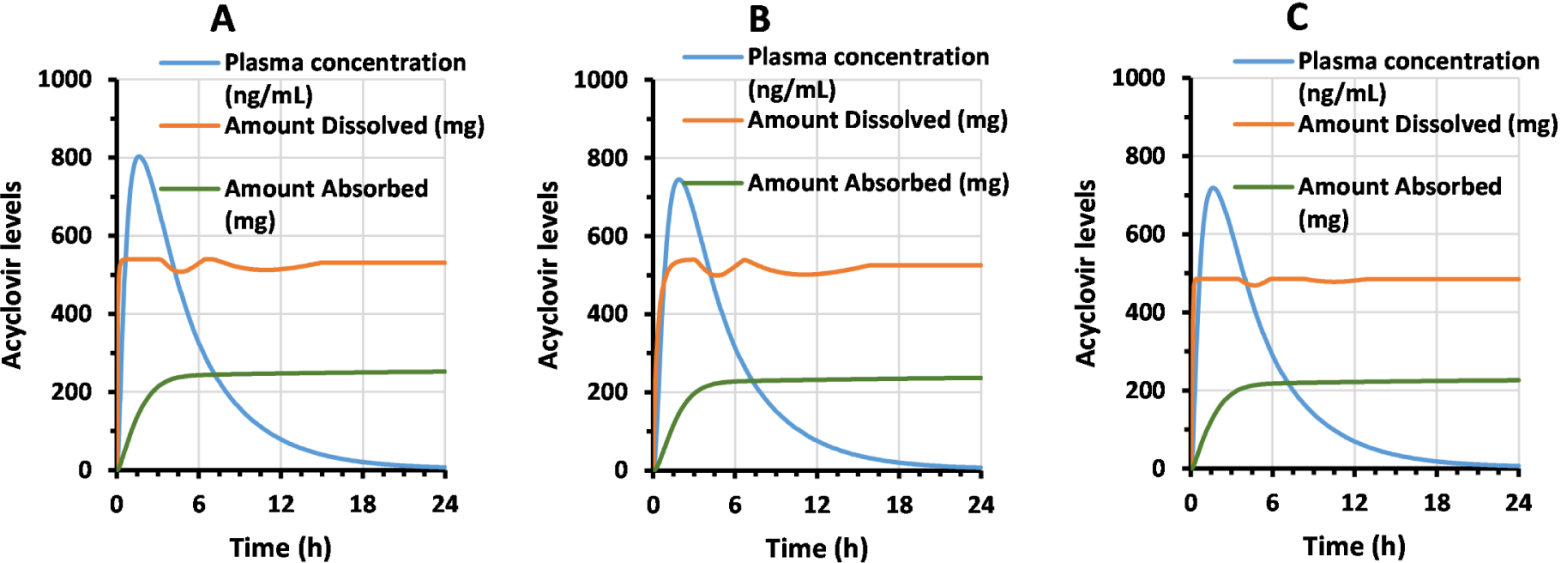
Simulations of acyclovir plasma concentrations (blue), amount dissolved (orange), and amount absorbed (green) versus time. Panels A and B show simulations with the reference and product A, respectively, using the 150 mL/125 rpm method. Panel C shows simulations for the product A using the IPD method.

For product B, there is no evidence available on its bioequivalence status. Even though both the IPD and 150 mL/125 rpm methods agreed on the lack of bioequivalence for this product, only the former consistently predicted the lack of bioequivalence in every trial. Meanwhile, the 150 mL/125 rpm method predicted that product B would pass 40% of the VBE assays. This observation represents a risk for manufacturers who may decide on going to in vivo bioequivalence trials based on this result.

## 5 Conclusion

This article describes a model-informed approach to develop a universal in vivo predictive dissolution method for acyclovir IR tablets. The IPD conditions consisted of using the mini-vessel filled with 135 mL of hydrochloric acid, pH 2.0, 37 °C, and 150 rpm. Simulations confirmed the predictivity of these dissolution conditions by predicting not only single plasma profiles but also replicating in vivo bioequivalence outcomes through virtual bioequivalence trials.

## Supplementary Material


