# Supplementary material for: Development of a Universal In Vivo Predictive Dissolution Method for a Borderline BCS III/IV Drug Guided by Modeling and Simulations—Acyclovir as a Case Study
Source: Mol Pharm. 2025 Sep 22;22(10):6237–46. doi: 10.1021/acs.molpharmaceut.5c00981 (PMC12505258; doi:10.1021/acs.molpharmaceut.5c00981)
Supplement: Supplementary file 1 [file mp5c00981_si_001.pdf]

Development of a universal in vivo predictive dissolution method for a borderline BCS III/IV drug guided by modeling and simulations – Acyclovir as case study

Mauricio A. García[1*], Fernando Tapia[1], Benjamín Escares[1], Peter Langguth[2]

[1]Departamento de Farmacia, Escuela de Química y Farmacia, Facultad de Química y de Farmacia, Pontificia Universidad Católica de Chile, Santiago, 7820436, Chile.

[2]Departament of Biopharmaceutics and Pharmaceutical Technology, Johannes Gutenberg University Mainz, 55099, Mainz, Germany

*Corresponding author:

Email: magarci3@uc.cl

Phone: 56 95504 8410

Mail box: Vicuña Mackenna 4860, Campus San Joaquín, Macul, Santiago.

ABSTRACT:

Establishing in vivo predictive dissolution (IPD) conditions requires consideration of biopredictive aspects during dissolution. For acyclovir, lower dose strengths (200 and 400 mg) can dissolve completely in gastrointestinal fluids. However, luminal concentrations after administering the highest strength (800 mg) exceeds the BCS solubility threshold. Given its poor permeability, sink conditions are not granted for the highest strength dose. In this study, a universal IPD method for acyclovir tablets was developed using the mini-vessel/mini-paddle apparatus. Computational simulations in a physiologically-based pharmacokinetic (PBPK) model further guided the development. Apparatuses with different volumes and stirring conditions were explored and results served as input for the model. Dissolution of 800 mg acyclovir tablets in 900 ml medium largely overpredicted observed plasma profiles, due to poor resemblance of non-sink conditions in the lumen. Conversely, dissolution in the mini-vessel filled with 135 ml of HCl, pH 2.0, at 150 rpm, produced accurate predictions of plasma profiles, without affecting previous successful predictions with the lowest strength tablets. Furthermore, in-human and virtual bioequivalence studies confirmed the predictive potential of this method. Therefore, the aforementioned dissolution conditions can be considered as a universal IPD method for acyclovir immediate release tablets.

KEYWORDS:

in vivo predicted dissolution, virtual bioequivalence, PBPK, small volume dissolution testing, non-sink dissolution

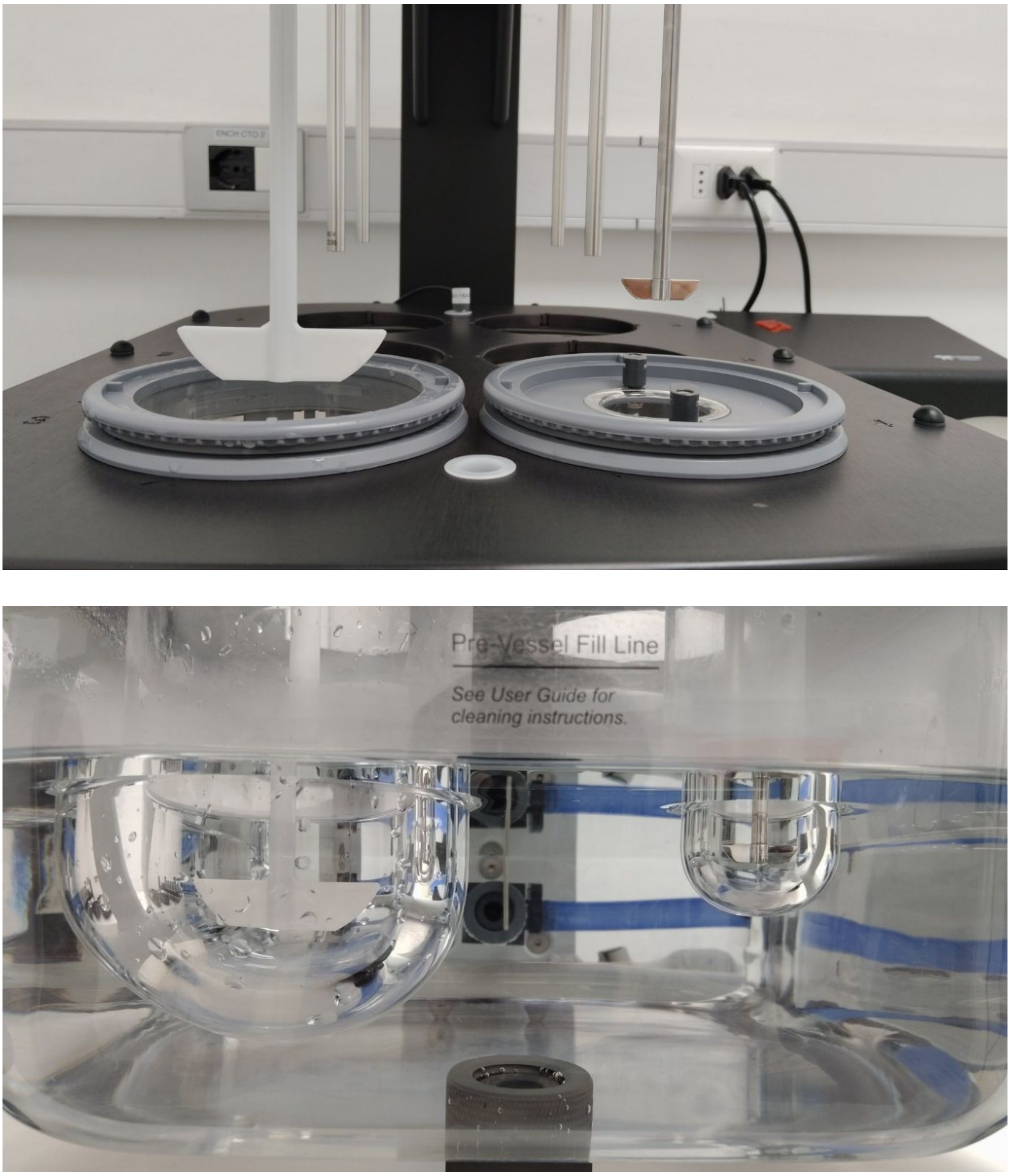

Fig S1. Comparison between conventional USP type II apparatus (left hand side) and mini-vessel (right hand side). Differences in paddles and vessels are shown in upper and lower panels, respectively.

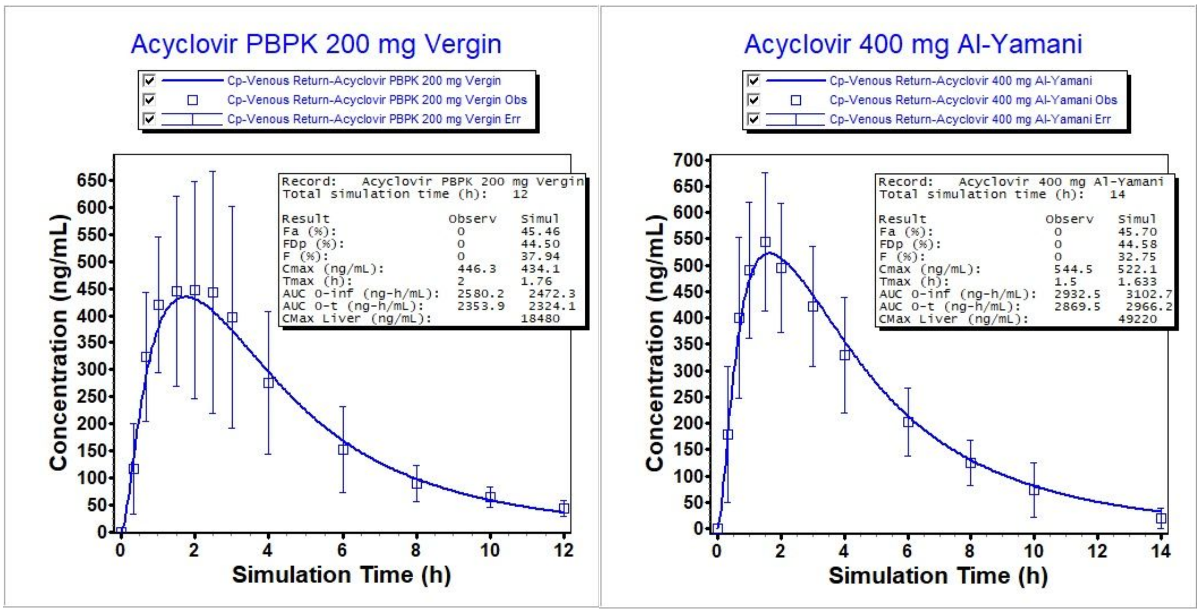

Fig S2. Model validation using data reported by Vergin et al., (left hand side panel) and Al-Yamani et al., (right hand side panel), with r^2=0.997 and 0.989, respectively. References are provided in the main text.

Table S1. Input parameters in the PBPK model.

Physicochemical parameter	Value	PBPK parameter		Value
log P	-1.57	SpecPStc (ml/s/ml)		$1.0x10^{-4}$
pKa base	2.27	OAT2	K_m (µM)	94
pKa acid	9.25		V_{max} (mg/s/mg Trans)	0.12
$S_{pH=5.8}$ (mg/ml)	2.33	OCT1	K_m (µM)	151
Fup (%)	84.6		V_{max} (mg/s/mg Trans)	0.035
Blood:plasma ratio	1.07	MATE 1	K_m (µM)	2,640
D (cm²/s, 10^{-6})	9.24		V_{max} (mg/s/mg Trans)	0.024
Human P_{eff} (cm/s, 10^{-4})	0.356			

For references, the reader is referred to García et al., 2021, and the main text. The OCT1 V_{max} value of 0.035 mg/s/mg Trans was selected to better match the population in which the study was conducted.

Table S2. Pharmacokinetic parameters from the bioequivalence trial

	Geometric mean (standard deviation)	
	Reference	Product A
C_{max} (ng /ml)	792.8 (315.0)	833.4 (306.2)
AUC_{0-t} (ng*h/ml)	3902.4 (1456.1)	3947.3 (1548.7)
AUC_{0-inf} (ng*h/ml)	4666.0 (2554.0)	4458.4 (1696.5)
T_{max} (h)	1.797 (0.749)	1.684 (0.647)

Table S3. Weibull parameters for each method.

	Reference	Product A	Product B	Product C
	Method: 135 ml/150 rpm			
α (time-scaling)	2.955×10^{-4}	0.0270	0.6102	0.0682
β (shape)	3.159	1.748	0.2635	1.058
W_{max} (fixed)	60.75%			
	Method: 150 ml/125 rpm			
α (time-scaling)	0.0953	0.3726	0.4070	0.0492
β (shape)	0.7735	0.8953	0.5567	0.7782
W_{max} (fixed)	67.5%			

W_{max} were calculated from solubility values and vessel volume as per equation (1).

Table S4. Prediction errors obtained with the IPD method (135 ml/150 rpm) for acyclovir 200 mg IR tablets used in the previous study (García et al., 2021).

Product	C_{max}	AUC
Reference	-2.91	-4.50
Product B	-3.36	-5.19
Average	-3.14	-4.84
